# Herpes simplex virus esophagitis in an immunocompromised patient: case report of an atypical presentation

**DOI:** 10.17843/rpmesp.2025.424.14674

**Published:** 2025-12-21

**Authors:** Gonzalo David Layza-Reyes, Camila Milagros León-Mejía, Eloy Maier Valencia Reyes, José Luis Villalva Luna, Gustavo Quispe-Villegas, Álvaro Bellido-Caparó

**Affiliations:** 1 Universidad Peruana Cayetano Heredia, Lima, Peru. Universidad Peruana Cayetano Heredia Universidad Peruana Cayetano Heredia Lima Peru; 2 Servicio de Gastroenterología, Hospital Cayetano Heredia, Lima, Peru. Servicio de Gastroenterología Hospital Cayetano Heredia Lima Peru; 3 Laboratorios de Investigación y Desarrollo, Universidad Peruana Cayetano Heredia, Lima, Peru. Universidad Peruana Cayetano Heredia Laboratorios de Investigación y Desarrollo Universidad Peruana Cayetano Heredia Lima Peru

**Keywords:** Esophagitis, Herpes Simplex Virus, Human Immunodeficiency Virus

## Abstract

We present the case of a 28-year-old male with diarrhea, headache, and significant weight loss. He was diagnosed with HIV in the AIDS stage (CD4: 3 cells/μL), *Cryptococcus neoformans* meningoencephalitis, and multisystemic tuberculosis. Given the persistence of diarrhea and negative coproparasitological studies, endoscopic studies were performed. Upper digestive endoscopy incidentally showed esophageal erosions coated with fibrin and punched-out ulcers; these findings were identified as herpetic esophagitis by histopathological and immunohistochemical analysis. Treatment with intravenous acyclovir was initiated, followed by oral administration for 14 days. This case highlights the importance of considering herpetic esophagitis in immunosuppressed patients, even in the absence of esophageal symptoms, and emphasizes the value of upper digestive endoscopy with biopsy in the comprehensive diagnosis of HIV/AIDS.

## INTRODUCTION

Infectious esophagitis ranks third among the causes of esophagitis, behind gastroesophageal reflux disease and eosinophilic esophagitis. This disease can result from bacterial, viral, fungal, or parasitic infections ^1^. It is more common in immunosuppressed patients, such as people with diabetes, cancer, or HIV, among others; although in rare cases, it can also affect immunocompetent individuals ^2^.

Important infectious agents include fungi (*Candida albicans*, *Aspergillus* spp., etc.), viruses (herpes simplex, cytomegalovirus, varicella zoster virus, etc.) and bacteria (*Staphylococcus aureus*, *spp.* species) ^3^. The probability of identifying a specific infectious agent varies according to the clinical situation, but in general, *Candida* spp. is the most common pathogen, especially in people with moderate immunosuppression ^3^. In patients with HIV, the incidence of infectious esophagitis increases as the CD4 cell count decreases ^4^. Furthermore, in those with severe immunosuppression, the spectrum of microorganisms responsible for this disease widens ^3^.

The clinical presentation of each infectious etiology is very similar ^5^. Typical symptoms include odynophagia, dysphagia, and retrosternal pain ^3^^,^^6^. Due to the similarity of the clinical and endoscopic presentations, esophageal tissue biopsy is considered a crucial procedure to reach the precise diagnosis of the responsible pathogen ^7^. Below, we present the clinical case of an immunocompromised patient incidentally diagnosed with esophagitis.

## CASE REPORT

### Patient Information

**Figure 1:**
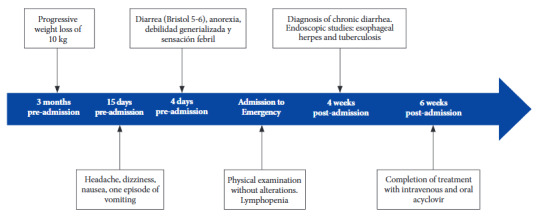
Timeline of the chronological report.

A 28-year-old male patient, with no significant medical history, came to the emergency department with a clinical presentation of 15 days of evolution, characterized by headache, dizziness, nausea, and one episode of vomiting with food contents. Four days before admission, diarrhea was added, with two liquid bowel movements daily (Bristol 5-6), without the presence of mucus or blood, in addition to anorexia, generalized weakness, and a feverish sensation. He also reported a weight loss of approximately 10 kg in the last three months (Figure 1).

### Clinical Findings

On admission physical examination, vital signs were within normal parameters. He did not present nuchal rigidity or other meningeal signs. The abdomen was soft, depressible, with preserved bowel sounds, not painful to superficial or deep palpation, with no masses or visceromegaly. The rest of the physical examination showed no pathological findings.

### Diagnostic Evaluation

The blood count showed lymphopenia (absolute lymphocyte count: 280 cells/μL), without other significant alterations. During his evaluation, he was diagnosed with *Cryptococcus neoformans* meningoencephalitis, HIV infection in the AIDS stage (CD4 count: 3 cells/μL; viral load: 15,200 copies/mL), and multisystemic tuberculosis, evidenced by a positive TB LAM Ag test in urine. During hospitalization, the diarrhea persisted for a period of four weeks, leading to the diagnosis of chronic diarrhea.

**Figure 2:**
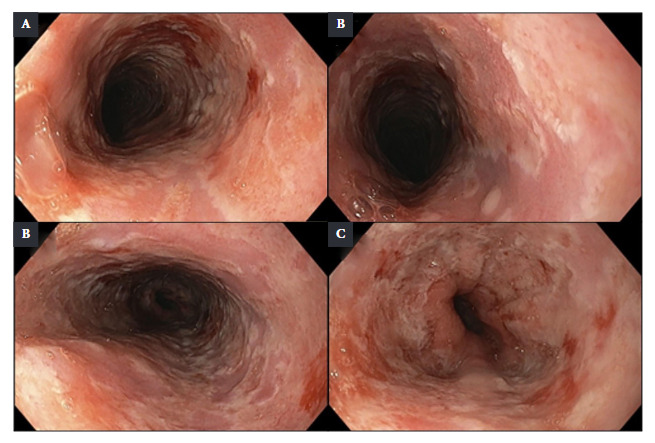
Upper digestive endoscopy findings. Multiple confluent erosions of 7 to 10 mm, covered with fibrin, affecting the entire circumference of the upper (A,B), middle (C) and lower thirds of the esophagus, including the esophago-gastric junction (D). Punched-out lesions and ulcers with flat edges and clean mucosal bases in the upper third, some confluent with each other (B).

**Figure 3:**
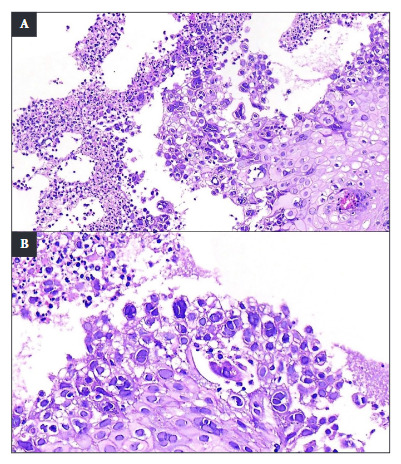
Esophageal biopsy stained with hematoxylin & eosin (H&E). Non-keratinized stratified squamous epithelium with inflammatory exudate is observed (x100) (A). At higher magnification, focal squamous cells were observed with ground-glass nuclei, multinucleation, nuclear molding, & chromatin margination, all suggestive of herpetic cytopathic changes (x200) (B).

**Figure 4:**
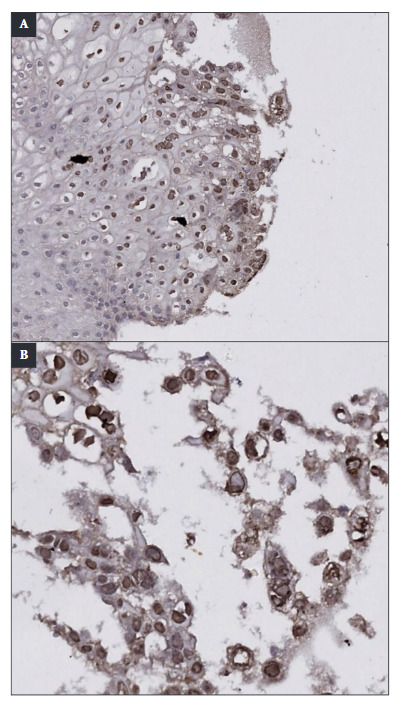
Immunohistochemistry with polyclonal antibodies for HSV-1 & HSV-2 was performed. At lower magnification (x100) (A) & at higher magnification (x200) (B), groups of epithelial cells with a nuclear stai-ning pattern were observed. It should be noted that the stained nuclei showed characteristic multinucleation & nuclear molding, both compa-tible with herpetic infection.

Since parasitological and coprological function tests were negative, endoscopic studies were indicated. Upper digestive endoscopy incidentally showed the presence of multiple confluent erosions, between 7 and 10 mm in diameter, coated by a layer of fibrin. These lesions compromised 100% of the esophageal circumference, extending from 20 cm distal to the dental arch to the esophagogastric junction. The lesions showed marked friability on contact (Figures 2A-D). Likewise, between 15 and 25 cm from the dental arch, punched-out ulcers with flat edges were identified, some of which converged on a clean mucosal base (Figure 2B). A histopathological analysis with hematoxylin and eosin (H&E) staining was performed, which revealed non-keratinized stratified squamous epithelium with an inflammatory exudate composed mainly of polymorphonuclear leukocytes and focal cells with herpetic cytopathic changes: multinucleation, nuclear molding, and chromatin margination. Nuclei with a ground-glass appearance and eosinophilic intranuclear inclusions (Cowdry type A inclusions) were also observed (Figure 3). Immunohistochemistry with polyclonal antibodies for Herpes Simplex Virus Type 1 (HSV-1) and Herpes Simplex Virus Type 2 (HSV-2) stained the infected squamous epithelial cells with the cytopathic changes previously described (Figure 4). Herpes Simplex Virus Esophagitis was diagnosed.

Lower digestive endoscopy showed no macroscopic alterations. However, a random colon biopsy revealed the presence of histiocytes containing acid-fast bacilli, positive for Ziehl-Neelsen staining, a finding compatible with intestinal tuberculosis, for which anti-tuberculosis treatment was initiated, with progressive improvement of symptoms observed.

### Therapeutic Intervention

Following the diagnosis of HSV esophagitis, antiviral treatment with acyclovir was initiated. During the first ten days, 250 mg of intravenous acyclovir were administered every 8 hours. Subsequently, it was switched to oral acyclovir at a dose of 300 mg every 8 hours.

### Follow-up and Outcomes

Three months later, additional serological studies with ELISA IgM/IgG immunoassay for HSV-1 and HSV-2 revealed only positive IgG values for HSV-1 (20.8 IU/ml). Due to severe immunosuppression and the presence of other opportunistic infections, the patient died months after starting hospitalization.

### Patient or Family Perspective

The relative states: “I am very grateful to the hospital and for the treatment my relative received. Despite the severity of his condition, we always saw that the medical team did everything possible to treat him in the best way. Although unfortunately my brother had multiple opportunistic infections and his immune system was very compromised.”

## DISCUSSION

Esophageal infection caused by HSV is uncommon and usually presents in immunocompromised patients, although it can rarely affect immunocompetent individuals. This type of infection is primarily associated with HSV-1, although cases caused by HSV-2 have also been described ^6^. The condition can arise from either a primary infection or a reactivation; the latter occurs when the virus spreads to the esophageal mucosa via the vagus nerve or by direct extension from an oropharyngeal infection to the esophagus ^8^^,^^9^. Therefore, herpetic esophagitis may present along with coexisting glossitis or oropharyngeal ulcers ^10^.

Symptoms include dysphagia, odynophagia, heartburn, nausea, chest pain, and hematemesis, abdominal pain, and anorexia ^3^^,^^6^^,^^11^^,^^12^. In immunocompromised patients, fever and retrosternal chest pain are frequent ^9^. The clinical presentation of each infectious etiology is very similar, which complicates the differential diagnosis when based solely on clinical characteristics ^5^. Clinical diagnosis becomes even more complex in patients with an atypical presentation, as in the case described, in which no esophageal symptoms were evident and, therefore, an infectious esophagitis was not initially suspected. However, this report demonstrates the need to always keep this entity within the differential diagnoses, especially in patients with significantly decreased CD4+ lymphocyte counts.

Diagnosis must be based on endoscopic findings confirmed with histopathological studies ^13^. Endoscopically, the differences between the various forms of infectious esophagitis are often minimal, as both *C. albicans* and HSV esophagitis can present with ulcerative or plaque lesions that are not easily distinguished from each other. Therefore, endoscopic findings are nonspecific, and their appearance may vary depending on the stage of the disease and the host’s immune response ^14^. According to the literature, in HSV esophagitis, lesions usually affect the lower third of the esophagus, initially presenting as vesicles which are rarely observed during endoscopy ^13^.

 Subsequently, the vesicles progress to punched-out ulcers that are usually less than 2 cm in diameter, round, and superficial, with an intermediate mucosa of normal appearance ^13^^,^^15^, mainly occupying the middle and lower third of the esophagus. The incidental endoscopic finding in our patient allowed the identification of lesions that diffusely compromised the entire esophagus, both transversely and longitudinally, including the upper third. This extensive involvement was striking due to the absence of clinical symptoms. Due to the magnitude of the lesions and their inconclusive characteristics, biopsies were taken for diagnostic confirmation.

 Biopsy samples should be taken at the edge of the ulcer, where viral cytopathic effects are most likely to be located ^16^. Histological findings include multinucleated giant cells, with ground-glass nuclei and eosinophilic inclusions (Cowdry type A inclusion bodies) that occupy up to half of the nuclear volume, nuclear molding, and chromatin margination ^6^^,^^11^. As the literature indicates, biopsy constitutes a fundamental tool to differentiate the etiology of infectious esophagitis. This examination should be performed when esophageal lesions are found in immunosuppressed patients, even if said lesions are characteristic of one pathogen, since, in patients with severe immunosuppression, there is the possibility that the condition is caused by concomitant agents. Thus, the identification of the etiologic agent is determining in establishing the appropriate treatment, given that therapeutic options differ according to the cause, being distinct for *Candida* spp., cytomegalovirus, and herpes simplex virus. In the presented case, immunohistochemistry and serological studies allowed confirmation of HSV-1 as the causative agent of the esophageal lesions.

Antiviral therapy primarily depends on the immune status. Unlike immunocompetent patients, in whom the infection is usually self-limiting, immunosuppressed patients require treatment to avoid lethal complications such as stenosis, tracheobronchial fistulas, upper gastrointestinal bleeding, or esophageal perforation. This must be with intravenous acyclovir, 250 mg every 8 hours for 7-10 days ^5^, the same dose and frequency that our patient received. In the case of the patient, the indication for endoscopy was due to chronic diarrhea, a common indication in immunosuppressed patients, and esophageal lesions were incidentally found. Thus, we highlight the importance of performing a systematic endoscopy that includes adequate visualization of the esophagus, in order to identify lesions that could go unnoticed due to the absence of symptoms, and, in case of any suspicion of a lesion, we recommend proceeding with a biopsy. A delay in diagnosis and treatment can lead to fatal complications.

### Implications for Public Health

This case presents a herpetic esophageal infection, an entity that, while constituting an expected manifestation in patients with severe immunosuppression, has no previous reports in the national literature. Likewise, its prevalence is considerably low compared to other etiologies of infectious esophagitis; in a study carried out in a third-level hospital, it was only detected in 2.6% of patients with HIV who presented esophageal symptoms over three years of follow-up ^17^.

In the context of infectious esophagitis, *Candida* spp. constitutes the main etiologic agent due to its high frequency; however, herpes simplex virus must also be considered, particularly given the sustained increase in the prevalence of HIV patients and the growing number of diagnoses in advanced stages. In fact, in Peru, it has been reported that 12.6% of new annual cases correspond to patients who present in the AIDS stage ^18^, as was the case with our patient. Furthermore, in Latin America, it is estimated that approximately 30% of people with HIV do not receive antiretroviral treatment, which translates into a three times higher AIDS-associated mortality compared to countries in Central and North America. In this scenario, HIV and opportunistic infections continue to represent a significant national public health problem ^19^.

This scenario underscores the importance of updating knowledge on the management of opportunistic infections, also incorporating entities of lower prevalence, such as herpetic esophagitis. This case offers high-quality endoscopic images and anatomopathological findings that contribute to the teaching field and emphasize the need to consider this infection in every immuno-compromised patient, since a delay in the diagnosis and timely treatment of herpetic esophagitis can lead to serious and even lethal complications.

Among the limitations of the case is the lack of molecular tests, such as viral PCR, which would have allowed more precise confirmation of the presence of the herpes simplex virus. Furthermore, the deficiencies of the national health system, especially the difficulty in performing endoscopic studies with biopsy in all immunosuppressed patients who require it, restricts the applicability of the recommendations of this case in the Peruvian context.

In conclusion, HSV esophagitis is a pathology that should be considered in immunocompromised patients, especially in those with low CD4+ cell counts, even in the absence of the classic symptoms described in the literature. Endoscopy is a valuable tool for diagnosis, as it allows direct visualization of esophageal lesions. If lesions are found, obtaining biopsies is essential to identify the causative pathogens and establish the appropriate specific treatment. In the context of the increase in HIV cases in our country, it is essential to maintain a broad diagnostic approach and we urge the incorporation of endoscopy with biopsy whenever possible in patients in the AIDS stage and performing a systematic visualization of the esophagus, even if the initial indication is different or the patient does not present esophageal symptoms.
