# Supplementary material for: Herpes simplex virus esophagitis in an immunocompromised patient: case report of an atypical presentation
Source: Rev Peru Med Exp Salud Publica. 2025 Dec 21;42(4):436–41. doi: 10.17843/rpmesp.2025.424.14674 (PMC12879978; doi:10.17843/rpmesp.2025.424.14674)
Supplement: Translation

# Translation: Esofagitis por virus del herpes simple en un paciente inmunocomprometido: reporte de caso de una presentación atípica

**Authors:** Gonzalo David Layza-Reyes, Camila Milagros León-Mejía, Eloy Maier Valencia Reyes, José Luis Villalva Luna, Gustavo Quispe-Villegas, Álvaro Bellido-Caparó

**Affiliations:** 1 Universidad Peruana Cayetano Heredia, Lima, Perú.; 2 Gastroenterology Service, Hospital Cayetano Heredia, Lima, Perú.; 3 Research and Development Laboratories, Universidad Peruana Cayetano Heredia, Lima, Perú.

**Keywords:** Esofagitis, Virus del Herpes Simple, Virus de Inmunodeficiencia Humana

## Abstract

Se presenta el caso de un varón de 28 años con diarrea, cefalea y pérdida de peso significativa. Fue diagnosticado con VIH en estadio SIDA (CD4: 3 células/μL), meningoencefalitis por *Cryptococcus neoformans* y tuberculosis multisistémica. Ante la persistencia de diarrea y estudios coproparasitológicos negativos, se realizaron estudios endoscópicos. La endoscopia digestiva alta mostró incidentalmente erosiones esofágicas recubiertas de fibrina y úlceras en sacabocados, estos hallazgos fueron identificados como esofagitis herpética mediante análisis histopatológico e inmunohistoquímico. Se inició tratamiento con aciclovir por vía intravenosa, seguido de administración oral durante 14 días. Este caso resalta la importancia de considerar la esofagitis herpética en pacientes inmunosuprimidos, incluso en ausencia de síntomas esofágicos, y destaca el valor de la endoscopia digestiva alta con toma de biopsia en el diagnóstico integral del VIH/SIDA.

## INTRODUCCIÓN

La esofagitis infecciosa ocupa el tercer lugar entre las causas de esofagitis, por detrás de la enfermedad por reflujo gastroesofágico y la esofagitis eosinofílica. Esta enfermedad puede ser resultado de infecciones bacterianas, virales, fúngicas o parasitarias ^1^. Es más común en pacientes inmunosuprimidos, como personas con diabetes, cáncer o VIH, entre otros; aunque en casos poco frecuentes también puede afectar a individuos inmunocompetentes ^2^.

Los agentes infecciosos importantes incluyen hongos (*Candida albicans*, *Aspergillus* spp., etc), virus (herpes simple, citomegalovirus, virus varicela zóster, etc.) y bacterias (*Staphylococcus aureus*, especies de spp.) ^3^. La probabilidad de identificar un agente infeccioso específico varía según la situación clínica, pero en general, *Candida* spp. es el patógeno más común, especialmente en personas con inmunodepresión moderada ^3^. En pacientes con VIH, la incidencia de esofagitis infecciosa se incrementa a medida que desciende el recuento de células CD4 ^4^. Además, en aquellos con inmunosupresión severa, el espectro de microorganismos responsables de esta enfermedad se amplía ^3^.

La presentación clínica de cada etiología infecciosa es muy similar ^5^. Los síntomas típicos incluyen odinofagia, disfagia y dolor retroesternal ^3^^,^^6^. Debido a la similitud de las presentaciones clínicas y endoscópicas, la biopsia del tejido esofágico se considera un procedimiento crucial para alcanzar el diagnóstico preciso del patógeno responsable ^7^. A continuación, se presenta el caso clínico de un paciente inmunosuprimido diagnosticado de manera incidental con esofagitis.

## REPORTE DE CASO

### Información del paciente

Paciente masculino de 28 años, sin antecedentes médicos de importancia, acude al servicio de emergencia por un cuadro clínico de 15 días de evolución, caracterizado por cefalea, mareos, náuseas y un episodio de vómito con contenido alimentario. Cuatro días antes del ingreso se añadió diarrea, con dos deposiciones líquidas diarias (Bristol 5-6), sin presencia de moco ni sangre, además de anorexia, debilidad generalizada y sensación febril. Refiere, además, una pérdida ponderal de aproximadamente 10 kg en los últimos tres meses (Figura 1).

**Figura 1:** Línea de tiempo del relato cronológico.

### Hallazgos clínicos

Al examen físico de ingreso, los signos vitales se encontraron dentro de parámetros normales. No presentó rigidez de nuca ni otros signos meníngeos. El abdomen era blando, depresible, con ruidos hidroaéreos conservados, no doloroso a la palpación superficial ni profunda, sin masas ni visceromegalias. El resto del examen físico no mostró hallazgos patológicos.

### Evaluación diagnóstica

En el hemograma se evidenció linfopenia (recuento absoluto de linfocitos: 280 células/μL), sin otras alteraciones significativas. Durante su evaluación se diagnosticó meningoencefalitis por *Cryptococcus neoformans*, infección por VIH en estadio SIDA (recuento de CD4: 3 células/μL; carga viral: 15 200 copias/mL) y tuberculosis multisistémica, evidenciada por prueba TB LAM Ag positiva en orina. Durante la hospitalización, la diarrea persistió por un periodo de cuatro semanas, lo que condujo al diagnóstico de diarrea crónica.

Dado que los exámenes parasitológicos y coprofuncionales resultaron negativos, se indicaron estudios endoscópicos. La endoscopia digestiva alta evidenció de manera incidental la presencia de múltiples erosiones confluentes, de entre 7 y 10 mm de diámetro, recubiertas por una capa de fibrina. Estas lesiones comprometían el 100% de la circunferencia esofágica, extendiéndose desde los 20 cm distales a la arcada dentaria hasta la unión esofagogástrica. Las lesiones mostraban marcada friabilidad al contacto (Figuras 2A-D). Asimismo, entre los 15 y 25 cm de la arcada dentaria, se identificaron úlceras de bordes planos con morfología en sacabocado, algunas de las cuales confluían sobre una base mucosa limpia (Figura 2B). Se realizó un análisis histopatológico con tinción de hematoxilina y eosina (H&E) donde reveló un epitelio escamoso estratificado no queratinizado con un exudado inflamatorio compuesto principalmente por leucocitos polimorfonucleares y células focales con cambios citopáticos herpéticos: multinucleación, moldeamiento nuclear y marginación de la cromatina. También se observaron núcleos con aspecto de vidrio deslustrado e inclusiones intranucleares eosinofílicas (inclusiones de Cowdry tipo A) (Figura 3). La inmunohistoquímica con anticuerpos policlonales para virus del herpes simple tipo 1 (VHS-1) y virus del herpes simple tipo 2 (VHS-2) tiñó las células epiteliales escamosas infectadas con los cambios citopáticos descritos anteriormente (Figura 4). Se diagnosticó esofagitis por virus del herpes simple.

**Figura 2:** Hallazgos de endoscopia digestiva alta. Múltiples erosiones confluentes de 7 a 10 mm, cubiertas de fibrina, que afectan toda la circunferencia de los tercios superior (A,B), medio (C) e inferior del esófago, incluyendo la unión esofagogástrica (D). Lesiones en sacabocados y úlceras con bordes planos y bases mucosas limpias en el tercio superior, algunas confluyentes entre sí (B).

**Figura 3:** Biopsia esofágica teñida con hematoxilina y eosina (H&E). Se observa un epitelio escamoso estratificado no queratinizado con exu-dado inflamatorio (x100) (A). A mayor aumento se observaron células escamosas focales con núcleos en vidrio deslustrado, multinucleación, moldeamiento nuclear y marginación de la cromatina, todos sugestivos de cambios citopáticos herpéticos (x200) (B).

**Figura 4:** Se realizó inmunohistoquímica con anticuerpos policlonales para VHS-1 y VHS-2. A menor aumento (x100) (A) y a mayor aumento (x200) (B) se observaron grupos de células epiteliales con un patrón de tinción nuclear. Cabe destacar que los núcleos teñidos mostraron mul-tinucleación y moldeo nuclear característicos, ambos compatibles con infección herpética.

La endoscopia digestiva baja no evidenció alteraciones macroscópicas. Sin embargo, una biopsia de colon aleatoria reveló la presencia de histiocitos que contenían bacilos ácido-alcohol resistentes, positivos a la tinción de Ziehl-Neelsen, hallazgo compatible con tuberculosis intestinal por lo que se inició tratamiento antituberculoso, observándose mejoría progresiva de los síntomas.

### Intervención terapéutica

Tras el diagnóstico de esofagitis por VHS, se inició tratamiento antiviral con aciclovir. Durante los primeros diez días, se administraron 250 mg de aciclovir intravenoso cada 8 horas. Posteriormente, se cambió a aciclovir vía oral a dosis de 300 mg cada 8 horas. 

### Seguimiento y resultados

Tres meses después, estudios serológicos adicionales con inmunoensayo ELISA IgM/IgG para VHS-1 y VHS-2 revelaron únicamente valores positivos de IgG para VHS-1 (20,8 UI/ml). Debido a la inmunosupresión severa y la presencia por otras infecciones oportunistas, el paciente falleció meses después de iniciar su hospitalización.

### Perspectiva del paciente o familiar

El familiar refiere: «Estoy muy agradecido con el hospital y con el tratamiento que recibió mi familiar. A pesar de la gravedad de su estado, siempre vimos que el equipo médico hizo todo lo posible por tratarlo de la mejor manera. Aunque lamentablemente mi hermano tenía múltiples infecciones oportunistas y su sistema inmunológico estaba muy comprometido».

## DISCUSIÓN

La infección esofágica causada por VHS es poco común y suele presentarse en pacientes inmunodeprimidos, aunque rara vez puede afectar a individuos inmunocompetentes. Este tipo de infección se asocia principalmente con el VHS-1, aunque también se han descrito casos causados por el VHS-2 ^6^. La afección puede surgir tanto de una infección primaria como de una reactivación; esta última ocurre cuando el virus se propaga a la mucosa esofágica a través del nervio vago o por extensión directa desde una infección orofaríngea al esófago ^8^^,^^9^. Por lo tanto, la esofagitis herpética puede presentarse junto con glositis o úlceras orofaríngeas coexistentes ^10^.

Los síntomas incluyen disfagia, odinofagia, pirosis, náuseas, dolor torácico y hematemesis, dolor abdominal y anorexia ^3^^,^^6^^,^^11^^,^^12^, en pacientes inmunodeprimidos, la fiebre y el dolor torácico retroesternal son frecuentes ^9^. La presentación clínica de cada etiología infecciosa es muy similar, lo que dificulta el diagnóstico diferencial cuando se basa únicamente en las características clínicas ^5^. El diagnóstico clínico se torna aún más complejo en pacientes con presentación atípica, como en el caso descrito, en el que no se evidenció sintomatología esofágica y, por tanto, no se sospechó inicialmente una esofagitis infecciosa. Sin embargo, este reporte evidencia la necesidad de mantener siempre esta entidad dentro de los diagnósticos diferenciales, especialmente en pacientes con recuentos de linfocitos CD4+ significativamente disminuidos.

El diagnóstico debe basarse en hallazgos endoscópicos confirmados con estudios histopatológicos ^13^. Endoscópicamente, las diferencias entre las distintas formas de esofagitis infecciosa suelen ser mínimas, ya que tanto la esofagitis por *C. albicans* como por VHS pueden presentarse con lesiones ulcerativas o en placa que no se distinguen fácilmente entre sí. Por lo tanto, los hallazgos endoscópicos son inespecíficos y su aspecto puede variar según el estadio de la enfermedad y la respuesta inmunitaria del huésped ^14^. Según la bibliografía, en una esofagitis por VHS, las lesiones suelen afectar el tercio inferior del esófago, presentándose inicialmente como vesículas que rara vez se observan durante la endoscopia ^13^. Posteriormente, las vesículas progresan a úlceras en sacabocados que suelen tener menos de 2 cm de diámetro, son redondas y superficiales, con una mucosa intermedia de aspecto normal ^13^^,^^15^, ocupando principalmente el tercio medio e inferior del esófago. El hallazgo endoscópico incidental en nuestro paciente permitió identificar lesiones que comprometían de manera difusa todo el esófago, tanto en sentido transversal como longitudinal, incluyendo el tercio superior. Este compromiso extenso resultaba llamativo por la ausencia de sintomatología clínica. Debido a la magnitud de las lesiones y a sus características no concluyentes, se procedió a realizar la toma de biopsias para su confirmación diagnóstica.

Las muestras de biopsia deben tomarse en el borde de la úlcera, donde es más probable que se localicen los efectos citopáticos virales ^16^. Los hallazgos histológicos incluyen células gigantes multinucleadas, con núcleos vítreos e inclusiones eosinofílicas (cuerpos de inclusión tipo A de Cowdry) que ocupan hasta la mitad del volumen nuclear, moldeado nuclear y marginación de la cromatina ^6^^,^^11^. Como lo señala la literatura, la biopsia se constituye como una herramienta fundamental para diferenciar la etiología de la esofagitis infecciosa. Este examen debe realizarse cuando se encuentran lesiones esofágicas en pacientes inmunosuprimidos, incluso si dichas lesiones son características de un patógeno, ya que, en pacientes con inmunosupresión severa, existe la posibilidad de que el cuadro sea causado por agentes concomitantes. De este modo, la identificación del agente etiológico resulta determinante para instaurar el tratamiento adecuado, dado que las opciones terapéuticas difieren según la causa, siendo distintas para *Candida* spp*.*, citomegalovirus y virus herpes simple. En el caso presentado, los estudios de inmunohistoquímica y serológicos permitieron confirmar al VHS-1 como agente causante de las lesiones esofágicas.

La terapia antiviral depende principalmente del estado inmunitario. A diferencia de pacientes inmunocompetentes, en quienes la infección suele ser autolimitada, los pacientes inmunosuprimidos requieren tratamiento a fin de evitar complicaciones letales tales como estenosis, fistulas traqueobronquiales, hemorragia digestiva alta o perforación esofágica. Esta debe ser con aciclovir intravenoso, 250 mg cada 8 horas durante 7-10 días ^5^, misma dosis y frecuencia que nuestro paciente recibió. En el caso del paciente, la indicación de la endoscopia se debió a la diarrea crónica, una indicación común en pacientes inmunosuprimidos e incidentalmente, se hallaron lesiones esofágicas. De este modo, destacamos la importancia de realizar una endoscopia sistemática que incluya una adecuada visualización del esófago, con el fin de identificar lesiones que podrían pasar desapercibidas debido a la ausencia de síntomas y, ante cualquier sospecha de lesión, recomendamos proceder con la toma de biopsia. Un retraso en el diagnóstico y tratamiento puede llevar a complicaciones fatales.

### Implicancias para la salud pública

El presente caso expone una infección por herpes esofágico, entidad que, si bien constituye una manifestación esperada en pacientes con inmunosupresión severa, no cuenta con reportes previos en la literatura nacional. Asimismo, su prevalencia resulta considerablemente baja en comparación con otras etiologías de esofagitis infecciosa; en un estudio realizado en un hospital de tercer nivel, únicamente se detectó en el 2,6% de los pacientes con VIH que presentaron sintomatología esofágica a lo largo de tres años de seguimiento ^17^.

En el contexto de la esofagitis infecciosa, *Candida* spp. constituye el principal agente etiológico debido a su elevada frecuencia; no obstante, el virus herpes simple también debe ser considerado, particularmente ante el incremento sostenido de la prevalencia de pacientes con VIH y el creciente número de diagnósticos en estadios avanzados. De hecho, en el Perú, se ha reportado que el 12,6% de los nuevos casos anuales corresponden a pacientes que debutan en estadio SIDA ^18^, tal como se presentó nuestro caso. Asimismo, en Latinoamérica se estima que aproximadamente el 30% de las personas con VIH no recibe tratamiento antirretroviral, lo que se traduce en una mortalidad asociada al SIDA tres veces mayor en comparación con países de Europa Central y Norteamérica. En este escenario, el VIH y las infecciones oportunistas continúan representando un importante problema de salud pública nacional ^19^.

Este escenario subraya la importancia de actualizar los conocimientos en el manejo de infecciones oportunistas, incorporando también a entidades de menor prevalencia, como la esofagitis herpética. El presente caso ofrece imágenes endoscópicas y hallazgos anatomopatológicos de alta calidad que contribuyen al ámbito docente y enfatizan la necesidad de considerar esta infección en todo paciente inmunocomprometido, puesto que el retraso en el diagnóstico y tratamiento oportuno de la esofagitis herpética puede derivar en complicaciones graves e incluso letales.

Entre las limitaciones del caso se encuentra la falta de pruebas moleculares, como la PCR viral, que habrían permitido confirmar con mayor precisión la presencia del virus del herpes simple. Además, las deficiencias del sistema de salud nacional, especialmente la dificultad para realizar estudios endoscópicos con biopsia en todos los pacientes inmunosuprimidos que lo requieran, restringe la aplicabilidad de las recomendaciones de este caso en el contexto peruano.

En conclusión, la esofagitis por VHS es una patología que debe considerarse en pacientes inmunodeprimidos, especialmente en aquellos con recuentos bajos de células CD4+, incluso en ausencia de los síntomas clásicos descritos en la literatura. La endoscopia es una herramienta valiosa para el diagnóstico, ya que permite la visualización directa de lesiones esofágicas. En caso de encontrarse lesiones, la obtención de biopsias es fundamental para identificar los patógenos causantes y establecer el tratamiento específico adecuado. En el contexto del incremento de casos de VIH en nuestro país, resulta esencial mantener un abordaje diagnóstico amplio y exhortamos incorporar la endoscopía con toma de biopsias siempre que sea posible en pacientes en estadio SIDA y realizando una visualización sistemática del esófago, incluso si la indicación inicial es otra o el paciente no manifiesta síntomas esofágicos.
